# Polyphenols as Modulator of Oxidative Stress in Cancer Disease: New Therapeutic Strategies

**DOI:** 10.1155/2016/6475624

**Published:** 2015-11-16

**Authors:** Anna Maria Mileo, Stefania Miccadei

**Affiliations:** Regina Elena National Cancer Institute, 00144 Rome, Italy

## Abstract

Cancer onset and progression have been linked to oxidative stress by increasing DNA mutations or inducing DNA damage, genome instability, and cell proliferation and therefore antioxidant agents could interfere with carcinogenesis. It is well known that conventional radio-/chemotherapies influence tumour outcome through ROS modulation. Since these antitumour treatments have important side effects, the challenge is to develop new anticancer therapeutic strategies more effective and less toxic for patients. To this purpose, many natural polyphenols have emerged as very promising anticancer bioactive compounds. Beside their well-known antioxidant activities, several polyphenols target epigenetic processes involved in cancer development through the modulation of oxidative stress. An alternative strategy to the cytotoxic treatment is an approach leading to cytostasis through the induction of therapy-induced senescence. Many anticancer polyphenols cause cellular growth arrest through the induction of a ROS-dependent premature senescence and are considered promising antitumour therapeutic tools. Furthermore, one of the most innovative and interesting topics is the evaluation of efficacy of prooxidant therapies on cancer stem cells (CSCs). Several ROS inducers-polyphenols can impact CSCs metabolisms and self-renewal related pathways. Natural polyphenol roles, mainly in chemoprevention and cancer therapies, are described and discussed in the light of the current literature data.

## 1. Introduction

Many epidemiological studies suggest that diet particularly rich in fruits and vegetables have cancer preventive properties [1–3]. The beneficial effects of diet are attributable, at least in part, to polyphenols which have antitumour activities both in animal models and in humans [4, 5].

During the past few decades the growing interest in natural polyphenols has contributed to understanding these compounds in terms of their chemical and biological functions and beneficial effects on human health [6, 7]. With the advent of cellular, molecular experimental systems, and transgenic/knockout mice models, relevant advances in understanding the mechanisms involved in the action of polyphenols have been achieved. Most of the beneficial effects of natural polyphenols are considered to reflect their ability to scavenge-free radicals endogenously generated [8] or formed by radiation and xenobiotics [9]. However, some data in literature, suggest that the antioxidant properties of the phenolic compounds may not fully account for their chemopreventive effects [10]. Emerging evidence indicates that these polyphenols may also behave as prooxidants to initiate a reactive oxygen species mediated cellular DNA breakage and consequent cell death [11]. It has been reported that such a prooxidant mechanism is a result of redox-active microenvironment in cancer cells due to elevated levels in copper [12]. Copper is an important redox-active metal ion present in chromatin, closely associated with DNA bases and can be mobilized by metal chelating agents. Several studies have established that serum, tissue, and cellular copper levels in cancer patients are significantly elevated. Given that aberrant redox system is frequently observed in many tumour cells [13], it was hypothesized that polyphenols may selectively affect tumour cells behaviour based on their differential redox status [12].

The protective mechanisms that block the initiation of carcinogenesis can be defined as chemoprevention, a concept that was originally introduced by Wattenberg [14]. Interestingly, natural polyphenols could induce apoptotic cell death in preneoplastic or neoplastic cells through various growth inhibitory mechanisms as the activation of cytochrome c and caspases, the arrest of cell cycle, and the modulation of signalling pathways (NF-*κ*B, JAK/STAT) which result in the inhibition of tumour progression [15, 16].

Research on the anticancer activities of dietary polyphenols, identified new antitumour molecules that can be used in cancer prevention and treatment, both alone and in combination with current chemotherapy/radiotherapy [17–19].

Cellular senescence is a physiological process of irreversible cell-cycle arrest that contributes to various physiological and pathological processes of aging [20]. Replicative senescence (RS) is associated with telomere erosion after repeated cell divisions, whereas stress-induced premature senescence (SIPS) is a telomere-independent process and occurs in response to aberrant oncogenic signalling, oxidative stress, and DNA damage. Although senescent cells have irreversibly lost their capacity for cell division, they are viable and remain metabolically active [21, 22].

Induction of cellular senescence can be considered a relevant mechanism of tumour suppression. The concept of prosenescence therapy has emerged over the past few years as a novel therapeutic approach to treat cancers. Emerging evidence has demonstrated that therapy-induced senescence (TIS) is a critical mechanism through which many anticancer drugs inhibit tumour progression [23]. TIS may be viewed either as an independent approach to treat cancer cells or as a combined strategy with conventional chemo-/radiotherapy. In a neoadjuvant setting, prosenescence therapy could be used with traditional treatments in order to reduce tumour mass before surgery. Furthermore, the engagement of prosenescence as an adjuvant therapy could be helpful in reducing the statistical risk of cancer relapse.

Epigenetic alterations, such as DNA methylation, histone acetylation level, and gene expression miRNA-regulated cancer stem cells biology, and induction of premature senescence in tumour cells have been identified as relevant anticancer features of many dietary polyphenolic compounds [24–28]. Increasing data from both cancer epidemiology and experimental attempts support the bright future of polyphenols as epigenetic modulators, prosenescence inducers, and cancer stem cells metabolism regulators in new anticancer approaches.

In this review, we will discuss the current progress in the study of polyphenols as very promising tools for the management of cancer prevention and treatment.

## 2. Oxidative Stress and Cancer

### 2.1. Cellular Transformation Mechanisms Mediated by ROS

Cancer is currently one of the most deadly diseases worldwide. According to a report by the World Health Organization (WHO) (http://who.int/cancer/en) 8.2 million people died of cancer in 2012; however 30% of cancer can be prevented and some of the most common cancers such as breast, colorectal, and cervical cancer are curable if treated promptly. Among many factors that cause cancer, oxidative stress is one of the most important and well-studied event that gives rise to the conditions leading to tumour onset and progression [29].

It has been demonstrated that continuous inflammation may lead to a preneoplastic situation [30–32]. In chronically inflamed cells, the secretion of a large amount of reactive oxygen/nitrogen species (ROS/RNS) recruits more activated immune cells, which leads to the amplification of dysregulated processes and eventually to a preneoplastic condition. If the amount of cellular ROS/RNS produced is high enough to overcome endogenous antioxidant response, an irreversible oxidative damage to nucleic acids, lipids, and proteins may cause genetic and/or epigenetic alterations leading to the dysregulation of oncogenes and tumour suppressor genes. Hence, the oxidative stress and chronic inflammation processes are tightly coupled and the failure to block these processes could result in genetic/epigenetic changes that drive the initiation of carcinogenesis [33]. Furthermore, several studies have shown that oxidative stress affects several signalling pathways associated with cell proliferation [34]. Among them, the epidermal growth factor receptor signalling pathway (EGFR) can be mentioned and key signalling proteins, such as the nuclear factor erythroid 2-related factor 2 (NRF2), Ras/Raf, the mitogen activated protein kinases (MAPKs) ERK1/2, and MEK, phosphatidyl inositol 3-kinase (PI3K), phospholipase C, and protein kinase C are affected by oxidative stress [35–37]. Moreover, ROS alter the expression of the p53 suppressor gene that is a key factor in apoptosis. Thus, oxidative stress causes changes in gene expression, cell proliferation, and apoptosis and plays a significant role in tumour initiation and progression [37–40].

**Figure 1:**
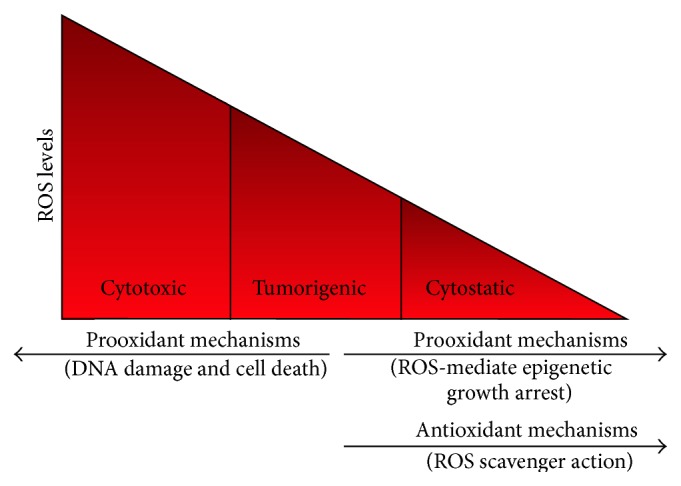
Dual prooxidant role of ROS level in cancer cells. Prooxidant mechanisms associated with different cellular ROS levels: high levels could induce DNA damage and cell death whereas low levels could induce epigenetic alterations and senescence-like growth arrest. In the figure the classical role of ROS scavengers as antioxidants is also reported.

Intriguingly, it is believed that, on the one hand, ROS contribute to the carcinogenesis, but, on the other hand, excessive amounts of ROS may act as cellular toxicants which lead to cancer cell growth arrest, apoptosis, or necrosis [41] (Figure 1). It is speculated that the malignant cells which are under increased level of oxidative stress would be more vulnerable to further ROS attack [42].

The evaluation of tissue redox status has great diagnostic potential in oncology. There are studies showing that low antioxidant status and increased oxidative stress levels are detected in cancer patients, even before oncology treatment starts [43]. Moreover, the redox status has a prognostic relevance for cancer therapy and could significantly contribute to the planning of an appropriate patient treatment regime. The conventional therapeutic strategy is based on drugs that increase ROS generation and induce apoptotic damage in cancer cells. However, this therapeutic approach has a serious disadvantage such as the development of various toxic side effects in normal tissues.

It has been reported [44] that normal cells compared to cancer cells show a low steady-state level of ROS and constant level of reducing equivalents. The different redox status of normal and cancer cells allows the use of this parameter for the design of new promising therapeutic strategies based on the regulation of redox signalling.

There is preliminary evidence suggesting that certain antioxidant supplements may reduce adverse cancer therapeutic reactions including neurotoxicity, asthenia, stomatitis/mucositis, and weight loss [45]. Significant reductions in toxicity may alleviate dose-limiting side effects so that more patients are able to complete prescribed chemo-/radiotherapy regimens successfully, suggesting an improved therapeutic index.

Furthermore, from a chemopreventive point of view, antioxidants have been shown to play an important role. Many epidemiological studies concluded that people who eat more vegetables, fruits, and other types of food rich in phenolic antioxidants may have a lower risk of developing some types of cancer. It is well established that the Mediterranean dietary pattern has beneficial effects on the prevention of cancer incidence and mortality. The Mediterranean diet is characterized by high antioxidant content capable of affecting inflammatory progress, cell cycle, proliferation and apoptosis process, and gene expression modulation [3, 46].

### 2.2. Oxidative Stress: Role of Epigenetic Alterations in Cancer

Since 1940, epigenetics has been defined as heritable changes in gene expression without changes in the DNA sequence and described the interactions between the genome and the environment that leads to the formation of the phenotype [47, 48]. Traditional epigenetic changes such as DNA methylation and histone modifications are able to affect gene expression mostly by interfering with the accessibility of transcription factors with DNA or may lead to structural rearrangement of chromatin thus promoting the expression of particular genes. Recent evidence has shown the association of altered expression of noncoding RNAs in general and microRNAs (miRNAs) in particular with epigenetic modifications [25]. miRNAs are small RNA molecules, ~22 long nucleotides, that can negatively control their target gene expression at a posttranscriptional level. miRNAs bind to their target mRNAs and downregulate their stabilities and/or translation. Accumulating evidences have shown that epigenetic alterations can largely contribute to the carcinogenesis [49] and are considered a hallmark of cancer [50]. The onset and progression of cancer are driven not only by acquired genetic alterations but also epigenetic modifications of gene expression [51, 52]. In cancer cells, hypermethylation on certain promoter regions of tumour suppressor genes causes gene silencing, thereby blocking the expression of these pivot genes [53]. Oxidative stress and inflammatory damage play an important role in epigenetic reprogramming of expression of cytokines, oncogenes, and tumour suppressor genes, thereby setting up a ground for chronic inflammatory diseases and carcinogenesis [30, 31]. On the other hand, global hypomethylation of DNA causes global chromosome instability leading to various mutations and, eventually, to cancer progression [54]. Since epigenetic aberrations occur in early stages of cancer, interventional approaches targeting the epigenome have been proposed as preventive and therapeutic strategies. Unlike genetic defects, epigenetic modifications are reversible and represent a promising avenue for therapeutic intervention [55]. Current epigenetic therapies aim to reverse cancer-associated epigenetic changes and restore normal gene expression. In this regard, two groups of drugs are approved for treatment by the Food and Drug Administration (FDA) [56, 57]: DNA methyl transferase (DNMT) and histone deacetylase (HDAC) inhibitors. Although some of them have even shown promising results in clinical trials [58], epigenomic therapies have several challenges ahead. To this purpose, it is relevant to note that various new DNMT as well as HDAC inhibitors are under development [59]. A synergistic combination of epigenetic modifying agents, including miRNAs, may provide a clinically important reversal of epigenomic cancer states.

## 3. Advances and Novelty in Cancer Chemoprevention and Treatment: Role of Polyphenols

### 3.1. Epigenetic and Antioxidant Treatment in Cancer

In addition to the standard anticancer treatment options such as surgery and chemo-/radiotherapy, several natural polyphenols have been identified as having potential for cancer prevention [60] and treatment [61] (Table 1).

Within the last few years most of the studies reporting on polyphenols have focused on their antioxidant properties [62]. In addition to their antioxidant ability to prevent damage caused by oxidative stress, polyphenols exert some of their biological effects via chromatin remodelling and other epigenetic modifications [63]. The beneficial effects of polyphenols in cancer treatment can be linked to their ability to modulate, in a reversible manner, epigenetic mechanisms involved in tumorigenesis leading to gene expression activation or silencing [64]. Many polyphenols are reported to regulate nuclear factor kappa B (NF-*κ*B) expression and chromatin remodelling through either activation or inhibition of epigenetic-related enzymes such as HDACs, histone acetyltransferases (HATs), and DNMTs [65]. In particular some polyphenols with antitumoural activity, such as genistein, phenethyl isothiocyanate, curcumin, sulforaphane, and resveratrol, act on the inhibition of deacetylation of histone proteins whereas other polyphenols, including epigallocatechin-3-gallate (ECGC), genistein, and curcumin, act on the inhibition of acetylation of histone proteins during epigenetic modifications [66]. Furthermore, dietary polyphenols, such as EGCG, genistein, lycopene, curcumin, and resveratrol, inhibit DNA methylation process by affecting DNA methyltransferase activity.

Cancer cells are distinguished by several distinct characteristics due to cumulative epigenetic changes of multiple genes and associated cell signalling pathways, some of which are linked to inflammation. Immune cells infiltrate tumours and are engaged in a cross talk with neoplastic cells; thus, inflammation might affect responses to cancer therapy [67]. Studies on a wide spectrum of various bioactive polyphenols that regulate multiple cancer-inflammation pathways and epigenetic cofactors exhibit low toxicity and are readily available [68]. The anti-inflammatory properties of many reported polyphenols are associated with their ability to induce HDAC activity [69].

Since several polyphenols can modulate both HDAC and HAT, there may be a common underlying mechanism. For instance, curcumin, a known antioxidant as well as a free radical, may regulate both acetylation and deacetylation through the modulation of oxidative stress. Rahman et al. [70] have shown that oxidative stress can induce NF-*κ*B pathway through the activation of intrinsic HAT activity, resulting in the expression of proinflammatory mediators, but it can also inhibit HDAC function.

The role of polyphenols in regulation of epigenetic pathways including sirtuin 1 (SIRT1) modulation has been investigated [71]. Sirtuins are a subclass of HDACs that have been shown to modify metabolism, inflammation, aging, or cellular apoptosis in many pathological processes. The epigenetic effect of SIRT1 is due to its ability to deacetylate many transcriptional factors such as p53, NF-*κ*B, forkhead box class O (FOX), and histone proteins [72].

Sulforaphane (SFN) is a bioactive polyphenol present in cruciferous vegetables such as broccoli, cabbage, and kale [73]. It has been previously shown that SFN induces the expression of phase-II detoxification enzymes [74] and expression of glutathione transferase in murine hepatocytes [75]. Furthermore SFN stimulates phase-II detoxification through activation of nuclear factor E-related factor 2 (NRF2) localized in the cytoplasm [76]. In response to oxidative stress, NRF2 translocates to the nucleus and binds to the antioxidant responsive element (ARE) promoting expression of antioxidant enzymes [77]. In a xenograft murine model, oral administration of SFN significantly reduces tumour size and increases apoptosis. These results indicated that SFN anticancer effects are exerted via inhibition of oxidative stress induced by NRF2-mediated pathways [77, 78]. In addition, SFN promotes anticancer effects through the inhibition of HDAC activity. For instance, in HCT116 colon cancer cell line, SFN inhibits HDAC activity in a dose-dependent manner [79]. A ten-week diet supplementation with SFN induces acetylation of histones in the ileum, colon, and prostate C57BL/6J mice tissues [80]. Moreover, sulforaphane-N-acetylcysteine (SFN-NAC) and sulforaphane-cysteine (SFN-Cys), two metabolites of SFN generated via the mercapturic acid pathway, may mediate the inhibitory effects on HDAC activity [80].

Isothiocyanate, such as phenethyl isothiocyanate (PEITC), has been shown to inhibit carcinogenic process by growth arrest of many types of cancer cells through induction of apoptotic pathway [81]. Treatment of human prostate cancer cell lines, with PEITC modulated histone acetylation and methylation pathways, in particular restored GSTP1 expression through demethylation of specific gene promoter and inhibited the activity of HDACs [82].

Curcumin, a polyphenol extracted from the most popular Indian turmeric spice (*Curcuma longa*), has antioxidant and anti-inflammatory properties which have been associated with multiple health benefits including cancer prevention [83]. In liver of lymphoma bearing mice long term effect of curcumin leads to prevention of cancer, by inducing phase-II antioxidant enzymes via activation of NRF2 signalling, restoration of tumour suppressor p53, and modulation of inflammatory mediators like TGF-*β* and COX2. These results suggest antioxidant and anti-inflammatory properties of curcumin [83].

Curcumin is a potential modulator of histones affecting both the HAT and HDAC enzyme activities [84]. Several* in vitro* studies, performed on cancer cell lines derived from various tissues, have demonstrated that curcumin has the potential to specifically downregulate p300/CBP HAT activity. In particular, such inhibition suppresses histone acetylation as well as acetylation of nonhistone protein like p53 [85]. Furthermore, curcumin exposure led to a significant reduction of histone acetylation via inhibition of HAT activity without changing HDAC levels in hepatoma cultured cells [84]. In hematopoietic cell lines, curcumin repressed the HAT activity of p300/CBP as well as the activity of various classes of HDACs, which in turn limits the proliferative capacity of cells and induces apoptosis [86]. Antitumour activity of curcumin has been also linked to its ability to modulate miRNA expression level in cancer cells. To this purpose, curcumin has shown to reduce the expression of the antiapoptotic protein Bcl-2 in a breast cancer cell line, MCF7, by upregulating miR-15a and miR-16 [87].

Green tea polyphenols are known to have high antioxidant properties and consequent beneficial functions, including anti-inflammation and cancer prevention. On the other hand, some studies have demonstrated their gastrointestinal toxicity when used at high doses, presumably due to their prooxidant properties [88]. Among green tea polyphenols, ECGC has been extensively studied. A treatment of high doses of this catechin may aggravate colon carcinogenesis in mice and induce hepatotoxicity in experimental animals and in humans as reported by epidemiological observations [88]. Importantly, it has been reported that EGCG can reduce cisplatin-mediated side effects treatment, in particular nephrotoxicity. Cisplatin, a cancer chemotherapeutic drug, induces kidney specific mitochondrial oxidative stress and impaired antioxidant defense enzyme activity. Treating mice with EGCG reduces cisplatin induced mitochondrial oxidative stress leading to an improved renal function compared to the counterparts. EGCG may be a potential and promising adjuvant agent for cisplatin cancer therapy [89]. Additionally these bioactive compounds are extensively studied from an epigenetically point of view. It has been shown that treatment on a human prostate cancer cell line altered DNA methylation levels and chromatin modelling and reduced the activity of Class I HDACs [90]. EGCG remarkably inhibits HAT activity, whereas other polyphenols derivatives, such as catechin, epicatechin, and epigallocatechin, exhibited low anti-HAT effects. EGCG acted as a HAT inhibitor and reduced the binding of p300/CBP to the promoter region of* interleukin-6* gene with an increased recruitment of HDAC3, which highlights the importance of the balance between HATs and histone deacetylases in the NF-*κ*B-mediated inflammatory signalling pathway [91]. Nandakumar and colleagues [92] demonstrated that EGCG-treatment of skin cancer cells modulated the levels of DNA methylation and histone modifications. These findings resulted in reexpression of tumour suppressor genes* p16* ^*INK4a*^ and* p21* ^*CIP/WAF1*^.

A combination of green tea polyphenols, a dietary DNA methyltransferase inhibitor and sulforaphane, a dietary histone deacetylase inhibitor leads to the epigenetic reactivation of silenced tumour suppressor genes such as* p21* ^*CIP/WAF1*^ and KLOTHO through active chromatin modifications in breast cancer cell lines [93]. These findings are relevant for understanding the potential of synergistical activity of polyphenol therapeutic combinations.

Treatment of various human cancer cell lines with EGCG caused a concentration and time-dependent reversal of hypermethylation of* p16* ^*INK4a*^,* p15*,* RARβ*,* MGMT*, and* hMLH1* genes [94, 95]. Furthermore, EGCG partially reversed the hypermethylation status of tumour suppressor gene* RECK* and enhanced the expression of RECK mRNA, which correlated with reduced expression of matrix metalloproteinases MMP-2 and MMP-9 involved in the invasive ability of cancer cells [96].

Aberrant promoter methylation of Wnt inhibitory factor-1 (WIF-1) is a fundamental mechanism of epigenetic silencing in human cancers. EGCG has been reported to directly reactivate the WIF-1, through the promoter demethylation in lung cancer cell lines [97].

EGCG modulated miRNAs in lung cancer and hepatocellular carcinoma where the expression of several miRNAs was changed [98, 99]. One of the upregulated miRNAs, miR16, specifically targets antiapoptotic protein Bcl-2 [99]. Altogether these pieces of data indicate that EGCG may be effective in different cancer cell types through different epigenetic pathways.

Coffee and tea polyphenols are also demethylating agents in human breast cancer cell lines where caffeic acid or chlorogenic acid inhibited DNMT1 activity, in a concentration-dependent manner [100].

Resveratrol (RV), a natural polyphenol found in blueberries, cranberries, nuts, red grapes, and wine, exerts anti-inflammatory and anticancer effects [101]. It has the ability to modulate signalling pathways that control cell growth, apoptosis, angiogenesis, and tumour metastasis processes [102]. Furthermore, RV is gaining attention for its antioxidant capabilities and influence on glucose metabolism. Oxidative stress and high glycolytic flux are common characteristics of cancer cells. It has been demonstrated that RV inhibits intracellular ROS level and suppresses cancer cell glycolytic metabolism [103].

Since anticancer biological activities are already demonstrated for RV and curcumin, to investigate the combined chemopreventive potential of these two polyphenols has been of great interest. It has been shown by Malhotra and colleagues [104] that curcumin and RV when supplemented in combination regulate drug-metabolizing enzymes and antioxidant enzymes, during lung carcinogenesis in mice.

RV activates the protein deacetylase SIRT1 leading to the formation of inactive chromatin and changes in gene transcription [103]. On the other hand, RV activates p300/CBP HAT that participates in the formation of an active chromatin structure [105]. Furthermore, Tili and his group [106] have shown that RV also inhibits oncogenic miRNAs while inducing tumour suppressor miRNAs. These multiple epigenetic alterations by RV exposure can partially explain the activation of some tumour suppressor genes.

In breast cancer, the tumour suppressor gene* BRCA1* is associated with lower levels of SIRTs expression. It has been reported that in* in vitro* and* in vivo* experimental models RV can increase the expression of BRCA1 by inhibiting Survivin expression and activating SIRT1. These findings suggest that resveratrol treatment serves as a potential strategy for targeted therapy for BRCA1-associated breast cancer [107]. Furthermore, RV in combination with black tea polyphenols suppresses growth and development of skin cancer in mice by inhibiting the MAPK and p53 pathways [108].

Isoflavones are compounds found in soy beans and act like estrogens. Among them, genistein and daidzein have gained the most research attention. Many studies have reported that genistein can be used as a chemopreventive agent in several types of cancers, especially for hormone-dependent breast cancer [109]. Genistein has been shown to bind both the estrogen receptor alpha (ER*α*) and the estrogen receptor beta (ER*β*). The ER*α*/ER*β* ratio is a prognostic marker for breast tumours, and ER*α* expression could indicate the presence of malignant tumours. It has been reported that in human breast cancer cell lines genistein effects depend on ER*α*/ER*β* ratio for oxidative stress regulation, mitochondrial functionality, and modulation of antioxidant enzymes, and sirtuins [110]. Genistein is also involved in the regulation of gene transcription by modification of epigenetic events including DNA methylation and histone modifications. Genistein has been shown to cause reversal of DNA hypermethylation and reactivated methylation-silenced genes, including tumour suppressor gene* p16* ^*INK4a*^ in human esophageal squamous carcinoma cell line [111]. In renal carcinoma, the cell tumour suppressor gene* BTG3* is transcriptionally downregulated. This inhibition is due to promoter CpG island methylation. The methylation-silenced* BTG3* gene can be reactivated by genistein treatment that causes CpG demethylation, inhibition of DNMT activity, and induction of active histone modifications [112].

Moreover, genistein treatment has shown the ability to modulate miRNAs expression level. For instance, in prostate cancer cells, genistein caused an increase of miRNA-1296 and accumulation of cells in the S phase of the cell cycle along with a significant downregulation of minichromosome maintenance gene (*MCM-2*), target of miRNA-1296 [113].

Quercetin, a dietary polyphenol present primarily in buckwheat and citrus and onions [114], is known to reduce intracellular ROS levels in various cell types by modulating detoxifying enzymes, such as superoxide dismutase 1 (SOD1) and catalase (CAT). Low concentration of quercetin attenuates the therapeutic effects of cisplatin and other antineoplastic drugs in ovarian cancer cells, by reducing ROS damage. The study concluded that quercetin supplementation during ovarian cancer treatment may detrimentally affect therapeutic response [115].

Quercetin activates SIRT1 deacetylase, through inhibition of HDAC and DNMT1, and has been shown to inhibit the cell cycle and induce apoptosis, thus suppressing tumour growth and angiogenesis [116].

Artichoke polyphenolic extracts had cytotoxic and apoptotic effects on colorectal cancer cells. It has been found that the proapoptotic* BAX* gene expression and a cell cycle inhibitor* p21* ^*CIP/WAF1*^ were induced in the presence of artichoke polyphenols [117]. Polyphenolic extracts from the edible part of artichoke (AEs) exhibited cancer cytotoxic activity on a human hepatoma cell line [118] as well as on other cell lines derived from various human tissues. It triggered apoptosis in a dose-dependent manner on a human breast cancer cell line without any effects on normal breast epithelial cell line. Furthermore, cell motility and invasion capabilities were remarkably inhibited by AEs treatment [119]. Furthermore AEs induce DNA hypomethylation and increase lysine acetylation levels in total proteins [28]. Importantly, the authors have shown that AEs have a prooxidant activity in breast cancer cells [28] and an antioxidant effect on normal hepatocytes [118].

From another point of view, chemopreventive polyphenols may indirectly modulate chromatin dynamics and epigenetic effects upon interference with global cancer metabolism [68]. To this purpose, the important role of sirtuins as principal intracellular mediators of the beneficial effect of the Mediterranean diet has been recently highlighted [46].

### 3.2. Therapy-Induced Senescence (TIS)

Cancer therapy has traditionally relied on cytotoxic treatment. This approach may produce complete cell death within neoplastic tissues; however such cancers often develop therapy-resistance and recur or progress to advanced primary and metastatic tumours. An alternative strategy to the cytotoxic treatment is the induction of cytostasis which disables the proliferation capacity of cells without inducing cancer cell death [120, 121] (Figure 1). This therapeutic approach could give an equivalent or prolonged survival with fewer or no side effects related to treatment toxicity in patients and may provide a more realistic goal for the chronic management of some cancers. To this purpose, a promising tool for generation of cytostasis is therapy-induced senescence (TIS) which promotes the induction of a permanent growth-arrest cellular phenotype with distinct morphological and biochemical characteristics [23]. The main features include development of a flattened and enlarged morphology* in vitro* and increased senescence-associated *β*-galactosidase activity in both cultured cells and tissues [122]. Unlike cells undergoing apoptosis in response to conventional cytotoxic drugs, senescent cells may persist almost indefinitely [121]. Several crucial genes, including tumour suppressors* p53* and* Rb*, have a well described growth inhibitory role [21, 123]. Cells with functional Rb and p53 appear more sensitive to stress and oncogene activities that stimulate senescence [124]. However, it is noteworthy from a therapeutic point of view that cancer cells lacking functional Rb, p53, and other tumour suppressor proteins display TIS responsiveness. Notably, in cancer cell lines lacking Rb and p53, doxorubicin induced senescence in more than 50% of cells without the direct involvement of these classic tumour suppressor genes [125].

Importantly, the combined activity of p53 and pRb could determine whether cells enter senescence or cell death pathways [126, 127]. However, the active role of these tumour suppressor proteins in senescence process is complex and actually not completely understood. Beside* Rb* and* p53*, several cell cycle involved genes including the cyclin-dependent kinase inhibitors (CDKIs)* p16* ^*INK4a*^,* p21* ^*CIP/WAF1*^, and* p27* [128] are active during senescence and promote senescent state when overexpressed in cancer cell lines. Overexpression of p21^CIP/WAF1^ can induce a senescence-like cell-cycle arrest, whereas depletion of p21^CIP/WAF1^ can delay senescence-associated arrest. Moreover, p16^INK4a^ acts in Rb pathway by inhibiting the activation of CDK4 and CDK6 which is the initial step of Rb phosphorylation [129]. The function of p16^INK4a^ is to keep Rb in its active, hypophosphorylated form, which blocks the expression of genes regulated by E2F transcription factors leading to a G1 cell-cycle arrest. Numerous studies have provided important insights into the p53/p21 and Rb/p16 pathways that promote cellular senescence: the first one is primarily responsible for senescence induced by telomere shortening or DNA damage; the second one is involved in mediated stress-induced premature senescence (SIPS).

The combination of prosenescence induction with already established treatment protocols in order to take into account the prosenescence approaches in the development of novel cancer therapies has been considered of interest. For instance, both neoadjuvant and adjuvant therapies have a more and more relevant role in the treatment of some neoplasias, including breast, prostate, and colon cancer, where such approaches significantly increase the disease-free survival and the overall survival of patients. In a neoadjuvant protocol, a prosenescence approach could be combined with traditional treatments in order to reduce tumour mass before surgery. Furthermore, senescence-inducing molecules such as natural compounds may be used in combination with radiotherapy in cancer patients who are not suitable for surgery because of their age or advanced stage of disease [130, 131]. Such a treatment may be expected to have two potentially positive outcomes. First, the induction of senescence itself may reduce tumour growth and trigger the immune system to clear senescence cells, contributing to reduction of the tumour mass. Second, since both senescence and apoptosis responses share key effector molecules (such as p53), the combination of prosenescence approaches with traditional chemo-/radiotherapeutic protocols may have the added effect of address cancer cells, which are en route to becoming senescent, toward apoptotic death.

A number of promising prosenescence agents are currently under consideration for cancer clinical management [132]. To this purpose, natural compounds targeting the epigenetic control of senescence are under investigations to develop additional prosenescence cancer therapeutic strategies [133, 134].

Several anticancer polyphenolic compounds from fruit and vegetables induce cellular growth arrest largely through the induction of a ROS-dependent premature senescence. Among them, 20(S)-ginsenoside Rg3 [135], a compound extracted from ginseng, at a subapoptotic concentration, caused senescence-like growth arrest and increased ROS production in chronically treated human glioma cells [135]. Furthermore, bisdemethoxycurcumin, a natural derivative of curcumin, suppresses human breast cancer cell proliferation by inducing oxidative stress senescence. A relevant role of ROS was also demonstrated for the phenethyl isothiocyanate-induction of apoptosis and senescence in tumours [136].

Polyphenolic extracts from the edible part of artichoke (AEs) have been shown to be potential chemopreventive and anticancer dietary compounds. High doses of AEs induce apoptosis and decrease the invasive potential of the human breast cancer cell line, MDA-MB231 [119]. Chronic and low doses of AEs treatment at sublethal concentrations suppress human breast cancer cell growth via the induction of premature senescence through epigenetic and ROS-mediated mechanisms [28]. In addition to the widely accepted antioxidant properties of the artichoke polyphenols [118], it has been demonstrated that one causative stimulus for senescence induction by chronic treatment of AEs is an increased level of reactive oxygen species. These results show a crucial role of ROS as effectors of polyphenol-induced prooxidant damage in cancer cells. To confirm this important contribution of ROS, the antioxidant NAC attenuates the effect of AEs on MDA-MB231. Importantly, the authors have shown that AEs have a prooxidant activity in breast cancer cells [28] and an antioxidant effect on normal hepatocytes [118]. Given that aberrant redox system is frequently observed in many tumour cells, the authors hypothesized that AEs may selectively inhibit the growth of tumour cells with little or no toxicity on normal cells based on their differential redox status.

Low doses treatment of RV exerts its anticancer and chemopreventive effects through the induction of premature senescence in lung cancer cells. This event correlates with increased DNA double strands breaks and ROS production through the upregulation of NAPDH oxidase-5 expression [137].

Furthermore, low doses of RV treatment arrested gastric cancer cells in the G1 phase and led to senescence instead of apoptosis which is initiated by high doses treatment [138]. The inhibitory effect of resveratrol on gastric cells was also verified* in vivo* using a nude mice xenograft model. RV exerted inhibitory activities on gastric development and significantly decreased the fraction of Ki67-positive cells in the nude mice tumour specimens. After the RV treatment, the induction of senescence and the changes in the expression of the regulators involved in the cell cycle and senescence pathways were similar to what was observed* in vitro*.

The propensity of tumour cells to undergo senescence in response to low and chronic exposure of RV treatment and to apoptosis with high doses of RV was confirmed in C6 rat glioma cells and further investigated in cooperation with quercetin. Chronically administered, RV and quercetin in subapoptotic doses can induce senescence-like growth arrest. These results suggested that the combination of these agents could be a good candidate treatment for glioma tumours [139].

## 4. Cancer Stem Cells: Potential Targets for Polyphenols

In the few last years, many studies have highlighted the existence of CSCs in most solid and nonsolid tumours, including brain, head and neck, breast, colon, and leukaemia among others [140–143]. These cells are considered responsible for tumour relapse and resistance to therapy [144–147]; thus novel therapeutic approaches, targeting the cancer stem cells pool, are under investigation [148]. Despite differentiated cancer cells, CSCs exhibit low ROS levels due to high expression of scavenger molecules, more efficient DNA repair responses, and promotion of glycolysis and autophagy [149, 150].

Recently, many strategies targeting cancer stem cells have been proposed, namely, (*a*) inhibiting their self-renewal ability and chemoresistance related pathways, (*b*) inducing their differentiation [151, 152], (*c*) targeting some of their cell-surface molecular markers [153], (*d*) impacting their energetic metabolism via inhibition of glycolysis [154] and/or by targeting mitochondria [155], and (*e*) designing miRNA-based strategies to block cancer stemness [156]. Theoretically in all tumours, cancer stem cells might reside within specific microenvironments distinguished by the presence of hypoxia [157], oxidative stress [158], chronic inflammation [159], and a peritumoural acidic pH [160]. Thus, many investigators have suggested that cancer can be overcome either by inhibiting the CSCs metabolisms or by targeting the surrounding cancer environment [161].

Novel anticancer strategies should be designed to selectively target cancer stem cells and to this purpose natural compounds might have a relevant role. We provide a revision of the most recent literature addressing the CSCs-regulation role of some of the most investigated polyphenols.

*(a) Role of Polyphenols in the Regulation/Inhibition of Cancer Stem Cells Self-Renewal*. It has been shown that polyphenols can impact cancer stem cells self-renewal related pathways, such as Wnt/*β*-catenin, Hedgehog, and notch [162]. In particular isothiocyanates (ITCs) have been described to have positive effects in the prevention of human tumours [163]. Beside several mechanisms of action, including activation of carcinogen-detoxifying enzymes, modulation of apoptotic pathway, cell-cycle arrest of cellular proliferation, and modulation of epithelial-mesenchymal transition (EMT), CSCs self-renewal suppression was reported, thus inhibiting oncogenic signalling pathways such as NF-*κ*B and STAT3 [164].

Between cruciferous family natural compounds, SFN has been demonstrated to be capable of targeting cancer stem cells in different types of cancer, by regulating pathways such as NF-*κ*B, Hedgehog, and Wnt/*β*-catenin also contributing to the induction of epithelial-mesenchymal transition. For these properties, SFN has been proposed as an adjuvant of chemotherapy in several preclinical studies [165]. Previous reports have demonstrated that SFN reduced the cancer stem cells population in human breast cancer cells as shown by decrease of aldehyde dehydrogenase (ALDH) + cells and reduction of primary mammospheres* in vitro* [166]. Furthermore, the antiproliferative property of SFN has been reported on pancreatic CSCs* in vitro* and* in vivo* models; such effect strongly depends on the activation of Hedgehog pathway for cancer stem cells self-renewal activity [167, 168].

Pancreatic CSCs studies carried out* in vitro* models, showed that quercetin, a polyphenol present in many fruit and vegetables [169], decreased ALDH1 activity, induced apoptosis, and decreased the expression of EMT-proteins. Whereas, in* in vivo* experiments, quercetin inhibited cancer stem cells-derived xenografts, reducing the expression of proliferation, stemness, and angiogenesis related genes [169].

Remarkably, quercetin effects were amplified in the presence of SFN, suggesting the importance to combine different polyphenols for designing synergistic anticancer strategies [169, 170].

The use of soy foods has been shown to be beneficial for the reduction of mammary tumour risk. The intake of these natural compounds was demonstrated to be beneficial for the modulation of body weight and adiposity associated with breast cancer both in humans and in animal models [171–174]. Moreover, human MCF-7 breast cancer cells cultured in a genistein-treated adipocytes conditioned medium generated a lower number of mammospheres [172].

*(b) Polyphenols Affecting Cancer Stem Cells Metabolism*. Cancer stem cells, like physiological stem cells, are characterized by a hyperglycolitic metabolism [175] and, in parallel, by a lowered mitochondrial respiration, compared to more differentiated cells within the tumour bulk [154, 176]. Thus a possible strategy to counteract CSCs could be to impair their metabolism either by inhibiting glycolysis or by forcing cancer stem cells into mitochondrial metabolism and oxidative phosphorylation [177]. To this purpose, many polyphenols have been shown to play a role in the regulation of cancer metabolism. Some plant derived polyphenols in relation to cancer cell metabolism are described. Genistein was shown to affect the pentose phosphate pathway (PPP), without modulating the synthesis of fatty acids in pancreatic adenocarcinoma cells [178]. Moreover, the green tea polyphenol, EGCG, is known to activate AMP-activated protein kinase (AMPK) in human breast cancer cells [179]. Activation of AMPK, a key actor in the control cellular energy status, cell cycle, protein synthesis, and cell viability, led to cell proliferation inhibition, upregulation of the CDK inhibitor* p21* ^*CIP/WAF1*^, downregulation of the mammalian target of rapamycin pathway, and suppression of the cancer stem cells population [179]. Moreover, polyphenols naturally present in extra olive oil have been shown to have anticancer effects by suppressing the expression of genes involved in both aerobic glycolysis (Warburg effect) and CSCs self-renewal [180].

Hyperglycolytic cancer stem cells have an increased basal level of ROS, although they result as being more vulnerable than physiological cells to a further increase in oxidative damage elicited by prooxidant polyphenol action [181]. One of these compounds is curcumin which, promoting ROS production, reducing the mitochondrial membrane potential, and inducing apoptotic pathways, leads to cell death in many cancer models [182, 183].

Cancer stem cells are localized within specific niches, normally characterized by the presence of lower oxygen tension (hypoxia), inflammation, oxidative stress, and a lower pH. Polyphenols can be exploited to regulate the CSC niche, by targeting signalling pathways that are implicated in the maintenance of tumour microenvironmental features. Among the polyphenols targeting hypoxia, pterostilbene, a stilbene isolated from blueberries, has been recently reported to have anticancer properties.

Breast cancer cell lines such as MCF-7 and MDA-MB231 were cocultured with tumour-associated macrophages, known to enhance malignancy promoting metastasis. In these experimental conditions, a large subpopulation of cancer stem cells, characterized by an increased level of HIF1*α*, *β*-catenin, Twist1, and NF-*κ*B and by a high ability to produce mammospheres, was present. By adding pterostilbene to the cell medium, the percentage of cancer stem cells was significantly reduced. The effects of such a polyphenol were confirmed* in vivo* experiments where tumorigenesis and metastasis were inhibited [184].

*(c) Polyphenols Targeting Proinflammation Signalling Pathways*. The presence of chronic inflammation could be a characteristic of the neoplastic niche [159]. Some polyphenols have been suggested to be potential therapeutic molecules to counteract chronic inflammatory status that eventually leads to several diseases including cancer [185]. Flavonoids, present in fruit and vegetables, are demonstrated to be suppressors of NF-*κ*B pathway, which is involved in inflammation, cellular transformation, tumour cell proliferation, invasion/metastasis, and angiogenesis [186]. Moreover, some carotenoids have been demonstrated to inhibit NF-*κ*B signalling and thus have anti-inflammatory and anticancer properties [187]. Recently, on a murine model of inflammation-triggered colon carcinogenesis, remarkable anti-inflammatory and antitumoural properties of glucosinolates, extracted from Brassicaceae, have been shown [188].

*(d) Polyphenols Regulating the Peritumoural Acidic pH*. Cancer stem cells extracellular microenvironment is often characterizated by an acidic status caused by CSCs metabolic dependence on aerobic glycolysis. Buffering the acidic cancer pH with the use of sodium bicarbonate inhibited tumour growth and cancer cell invasion in a preclinical animal model [189, 190]. To this purpose, high potassium intake coming from a diet rich in vegetables and fruits and a lowered consumption of animal proteins could be a natural strategy to neutralize cancer acidosis. An intriguing chemopreventive and therapeutic approach to raise pH could be the use of polyphenols such as genistein, EGCG, and RV in order to impair the cancer stem cells metabolism either by inhibiting aerobic glycolysis or by forcing them into oxidative phosphorylation, as previously described in this review.

Furthermore, several plant compounds have been shown to increase pH values by inhibiting proton pump activity and consequently elicited apoptosis in cancer [190]. This might represent another valid approach to counteract cancer cell growth.

## 5. Conclusions

**Figure 2:**
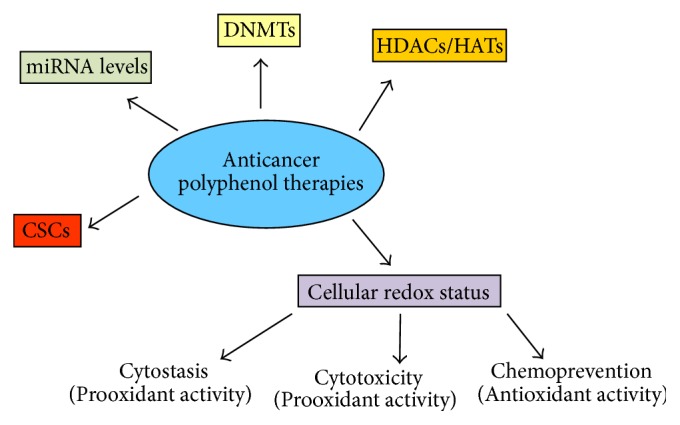
Targets of polyphenol anticancer therapies. Epigenetic pathways, cellular redox status, and cancer stem cells as therapeutic targets of polyphenol anticancer therapies as extensively discussed in the text. Depending on acute or chronic treatment a prooxidant activity may induce, respectively, high ROS-mediated cytotoxicity or low ROS-mediated cytostasis. According to the figure, several natural compounds as resveratrol, artichoke polyphenols, ginsenoside Rg-3, and quercetin induce a prooxidant apoptotic mechanism at high concentrations whereas low doses and chronic exposure trigger a ROS-epigenetic mediated cellular senescence.

Compared to normal cells, cancer cells have an increased rate of ROS production and have aberrant regulation mechanisms to deal with their particular redox status. ROS have a well-defined role in promoting and maintaining tumorigenicity indicating that dietary antioxidants have an active role in preventing or reducing tumorigenesis. On the other hand, high levels of ROS can also be toxic to neoplastic cells and can potentially induce cell death. Accumulating evidence shows that ROS levels in tumour cells are crucial for designing advanced therapies and future challenge in anticancer treatments. To this purpose, increasing knowledge from epidemiological and experimental data supports the bright future of natural polyphenols as anticancer tools [191–194] (Figure 2). The complex balance among cell proliferation, apoptosis, and senescence induced by polyphenols could be exploited therapeutically to improve the efficacy of conventional cancer treatment and to develop new antitumour strategies (Table 1).

Much attention is currently focused on the role of natural polyphenols on modulating intracellular ROS levels leading to epigenetic modifications of pivotal genes in tumorigenesis. It is important to stress that DNA methylation and posttranslational histone modifications are crucial actors in epigenomic landscape playing a relevant role in the structure and function of chromatin. Several polyphenols were demonstrated to interfere with enzymes driving the epigenetic alterations which modulate inflammation process that might hesitate in cancer. As such, it will be a challenge for future anti-inflammatory therapies to deeply evaluate the anticancer role of polyphenols as epigenetic modulators. However, there are some concerns that anticancer therapies with polyphenol regulators of DNMT and HAT/HDAC may suffer from a lack of specificity. To overcome this limitation, an alternative strategy may be to synergistically combine nonselective epigenetic treatments with low doses of conventional targeted therapies which lead to less toxicity comparing to a high dose standard treatments. Furthermore, microRNAs molecules are promising actors in the epigenetic combination therapies, as their target specificity may bridge the gap between genetic and epigenetic changes. To this purpose, natural polyphenols may indirectly modulate the epigenome by affecting levels of microRNAs which target specific epigenetic modifier enzymes.

## 6. Future Perspectives

The future of polyphenol-epigenomic therapy has several challenges ahead and it is a promising field for clinical cancer interventions.

In developing novel anticancer strategies, prosenescence has a relevant role. The current knowledge of senescence, as a major mechanism of tumour suppression as well as a determinant of the outcome of cancer treatment, leads to the concept of prosenescence therapy, which could be an important alternative or addition to conventional chemo-/radiotherapy. To this aim, prosenescence-polyphenols treatment may minimize toxicity and side effects of conventional therapies in cancer patients. On the other hand, some investigators suggest caution in the clinical management of this therapy because the induction of senescence might give rise to quiescent tumour cells, mainly cancer stem cells, which represent a potential niche for cancer recurrence. Thus, deeper understanding of the biological mechanisms responsible for cellular senescence is required in order to better characterize the role of polyphenols in prosenescence therapy for more efficient management of cancer treatment in the future.

The association of cancer stem cells and the resistance to chemo-/radiotherapy stimulate a critical consideration regarding the efficiency of prooxidant therapy on CSCs. Most conventional anticancer therapies are ineffective in killing this cell population. It is for this reason that there has been a growing interest to develop new strategies based on identifying agents able to directly target quiescent cancer stem cells. Since low ROS levels has been suggested to be critical for maintaining cellular stemness, an increase of these reactive species polyphenol-mediated might sensitize cancer stem cells to therapy. However, evaluating novel treatment approaches also require the development of assays or identification of biomarkers able to identify CSCs population in order to select and assess cancer patients.

## Figures and Tables

**Table 1:** Natural polyphenols as anticancer agents.

Bioactive components	Plants	Cancer models	Molecular mechanisms	References	Clinical trials references
Artichoke polyphenols	Artichoke	(i) Hepatocellular carcinoma and breast cancer cell lines	Apoptosis	[118, 119]	
		(ii) Breast cancer cell line	ROS/senescence, histone modifications, and DNA methylation	[28]	

Chlorogenic acid	Coffee	Breast cancer cell lines	DNA methylation	[100]	

Curcumin	Curcuma	(i) Lung cancer in mice	Apoptosis	[104]	Breast cancer Phase 2, [191] Colon rectal cancer Phase 1, [191]
		(ii) Pancreatic, prostate, and lung cancer cell lines	Histone modifications, DNA methylation, and miRNAs	[66]	

Daidzein	Soy	Breast cancer cell lines	Apoptosis	[109]	

Epigallocatechin-3-gallate	Green tea	(i) Hepatocellular carcinoma	miRNAs/apoptosis	[99]	Prostate cancer, [192] Leukaemia Phase 2, [193] Cancer prevention, [194]
		(ii) Skin cancer cell line	Histone modifications and DNA methylation	[92]	
		(iii) Breast cancer stem cells	Inhibition of mammosphere formation	[153]	

Genistein	Soy	(i) Prostate cancer cells and esophageal cell carcinoma	Histone modifications and DNA methylation	[111]	
		(ii) Renal carcinoma cell line	Histone modifications and DNA methylation	[112]	
		(iii) Breast cancer cell lines	Apoptosis	[109]	
		(iv) Breast cancer cell lines	Oxidative stress	[110]	

Ginsenoside Rg-3	Ginseng	Glioma cell lines	ROS/senescence	[135]	

Lycopene	Tomato	Breast cancer cells	DNA methylation	[66]	

Phenethyl isothiocyanate	Broccoli, cabbage, Brussels sprouts, and cauliflower	Prostate cancer cell lines	Histone modifications and DNA methylation	[82]	

Pterostilbene	Blueberries	Breast cancer stem cells	NF-*κ*B/miRNA488	[184]	

Resveratrol	Red grapes, cranberries, blueberries, and nuts	(i) Gastric cancer cell lines	Sirtuins/senescence	[138]	Colorectal cancer Phase 1, [191] Multiple myeloma Phase 2, [191] Melanoma Phase 1, [191]
		(ii) Lung cancer cell lines	ROS/senescence	[134]	
		(iii) Colon cancer cell lines	miRNAs	[106]	

Sulforaphane	Broccoli, cabbage, and kale	(i) Colon cancer cells	Histone modifications	[79]	
		(ii) Colon cancer in mice	Histone modifications	[80]	
		(iii) Breast cancer stem cells	Wnt/*β* catenin self-renewal pathway modulation	[166]	
		(iv) Pancreatic cancer stem cells	Hedgehog pathway activation	[165, 167]	

Quercetin	Onions, buckwheat, and citrus	(i) Oral carcinoma in hamster	Apoptosis, histone modifications, and DNA methylation	[116]	
		(ii) Pancreatic cancer cell lines and cancer stem cells	Apoptosis Inhibition self-renewal cell property	[169, 170]	

## Abbreviations

DNMTs: DNA methyl transferases
HDACs/HATs: Histone deacetylases/histone acetyltransferases
CSCs: Cancer stem cells
miRNAs: MicroRNAs.
